# Frequency of Spontaneous Neurotransmission at Individual Boutons Corresponds to the Size of the Readily Releasable Pool of Vesicles

**DOI:** 10.1523/JNEUROSCI.1253-23.2024

**Published:** 2024-02-21

**Authors:** Amelia J. Ralowicz, Sasipha Hokeness, Michael B. Hoppa

**Affiliations:** Department of Biology, Dartmouth College, Hanover, New Hampshire 03755

**Keywords:** neurotransmission, readily releasable pool, spontaneous release, synapse, vesicle

## Abstract

Synapses maintain two forms of neurotransmitter release to support communication in the brain. First, evoked neurotransmitter release is triggered by the invasion of an action potential (AP) across en passant boutons that form along axons. The probability of evoked release (*Pr*) varies substantially across boutons, even within a single axon. Such heterogeneity is the result of differences in the probability of a single synaptic vesicle (SV) fusing (Pv) and in the number of vesicles available for immediate release, known as the readily releasable pool (RRP). Spontaneous release (also known as a mini) is an important form of neurotransmission that occurs in the absence of APs. Because it cannot be triggered with electrical stimulation, much less is known about potential heterogeneity in the frequency of spontaneous release between boutons. We utilized a photostable and bright fluorescent indicator of glutamate release (iGluSnFR3) to quantify both spontaneous and evoked release at individual glutamatergic boutons. We found that the rate of spontaneous release is quite heterogenous at the level of individual boutons. Interestingly, when measuring both evoked and spontaneous release at single synapses, we found that boutons with the highest rates of spontaneous release also displayed the largest evoked responses. Using a new optical method to measure RRP at individual boutons, we found that this heterogeneity in spontaneous release was strongly correlated with the size of the RRP, but not related to Pv. We conclude that the RRP is a critical and dynamic aspect of synaptic strength that contributes to both evoked and spontaneous vesicle release.

## Significance Statement

Neurotransmitter is released through two mechanisms: AP-evoked and spontaneous vesicle fusion. It is unknown if some synapses specialize in either evoked or spontaneous release with an antagonistic relationship, or if the two forms of release coexist and have a cooperative relationship. We used a robust optical glutamate indicator to measure both forms of release at individual synapses. We found that the frequency of spontaneous release displays significant heterogeneity and is directly related to the size of the readily releasable pool of vesicles. This finding links both mechanisms of neurotransmitter release and suggests an important signaling mechanism to the postsynaptic neuron at individual synapses.

## Introduction

Brain function requires synaptic communication facilitated by vesicle fusion and the subsequent release of neurotransmitter. This release occurs in two ways: (1) evoked, triggered by the arrival of an action potential (AP), or (2) spontaneous, occurring in the absence of electrical stimulation. A number of studies suggest that at least some of the molecular mechanisms underlying each form of release are separate (Sara et al., 2005; Atasoy et al., 2008; Kavalali et al., 2011; Guzikowski and Kavalali, 2021), although far more molecular research has focused on evoked release (Rizo and Südhof, 2012). Evoked release is critical for rapid signaling between neurons within a circuit. Spontaneous release is less understood, but it has been found to influence synaptic development (Choi et al., 2014; Andreae and Burrone, 2015), maintenance (Sutton et al., 2004, 2006; Nosyreva et al., 2013), and plasticity (Sutton and Schuman, 2005; Jin et al., 2012; Banerjee et al., 2021; Sakimoto et al., 2022). Additionally, irregularities in spontaneous release are correlated with numerous neurological disorders (Hawkins, 2013; Wang et al., 2015; Nuriel et al., 2017; Alten et al., 2021). These reports suggest that spontaneous release may play an intrinsic role in synaptic physiology (Melom et al., 2013) and that the frequency of spontaneous release may vary across individual synapses. However, tools for making this determination at single presynaptic boutons did not exist until recently.

Spontaneous release is quantified as the frequency at which individual SVs fuse in the absence of stimulation. Interestingly, many changes in spontaneous release frequency have been reported in neurological disease models (Hawkins, 2013; Nuriel et al., 2017; Alten et al., 2021). In contrast with spontaneous neurotransmission, evoked release is quantified by the probability of vesicle fusion upon stimulation by an AP (*Pr*) (Südhof, 2013). *Pr* has been shown to vary substantially across individual boutons (Dobrunz and Stevens, 1997; Holderith et al., 2012; Ariel et al., 2013). Indeed, *Pr* has been shown to be controlled by two factors: (1) the number of docked vesicles, also known as the readily releasable pool (RRP), and (2) the probability that any one vesicle in the RRP will be released in response to a single AP, known as Pv (Ariel and Ryan, 2010). Docking is a multistep process during which SVs line up along the active zone (AZ) of the presynaptic membrane in a fusion-ready state (Südhof, 2013). An abundance of research demonstrates the functionality of this concept by showing that changes in RRP size affect evoked release (Ariel et al., 2013). Additionally, changes in RRP size are known to occur during various forms of plasticity as well as spine growth (Müller et al., 2012; Letellier et al., 2019; Fukaya et al., 2023). For any vesicle to undergo membrane fusion, evoked or spontaneous, it must be close enough to the plasma membrane, as is seen with vesicles in the RRP. This suggests that the number of vesicles within the RRP could influence both the *Pr* of evoked release as well as the frequency of spontaneous release.

Excitingly, the recent development of an improved fluorescent glutamate reporter, iGluSnFR3, provides low photobleaching rates and superior binding kinetics that avoid saturation from glutamate release (Aggarwal et al., 2023). This technique circumvents limitations previously encountered with both electrophysiological and fluorescent detection methods of neurotransmitter release. We took advantage of these features to measure properties of evoked release (Pv and RRP) as well as the frequency of spontaneous release at individual boutons in cultured hippocampal neurons. These measurements revealed a new relationship between the size of the RRP and the frequency of spontaneous release, unaffected by changes in Pv. These findings suggest a connection between seemingly disparate types of neurotransmitter release. Moreover, this suggests that spontaneous release transmits information about the RRP size, a critical aspect of presynaptic function, to the postsynaptic neuron.

## Results

### iGluSnFR3 can be used to detect evoked and spontaneous neurotransmitter release at individual presynaptic boutons

**Figure 1.**
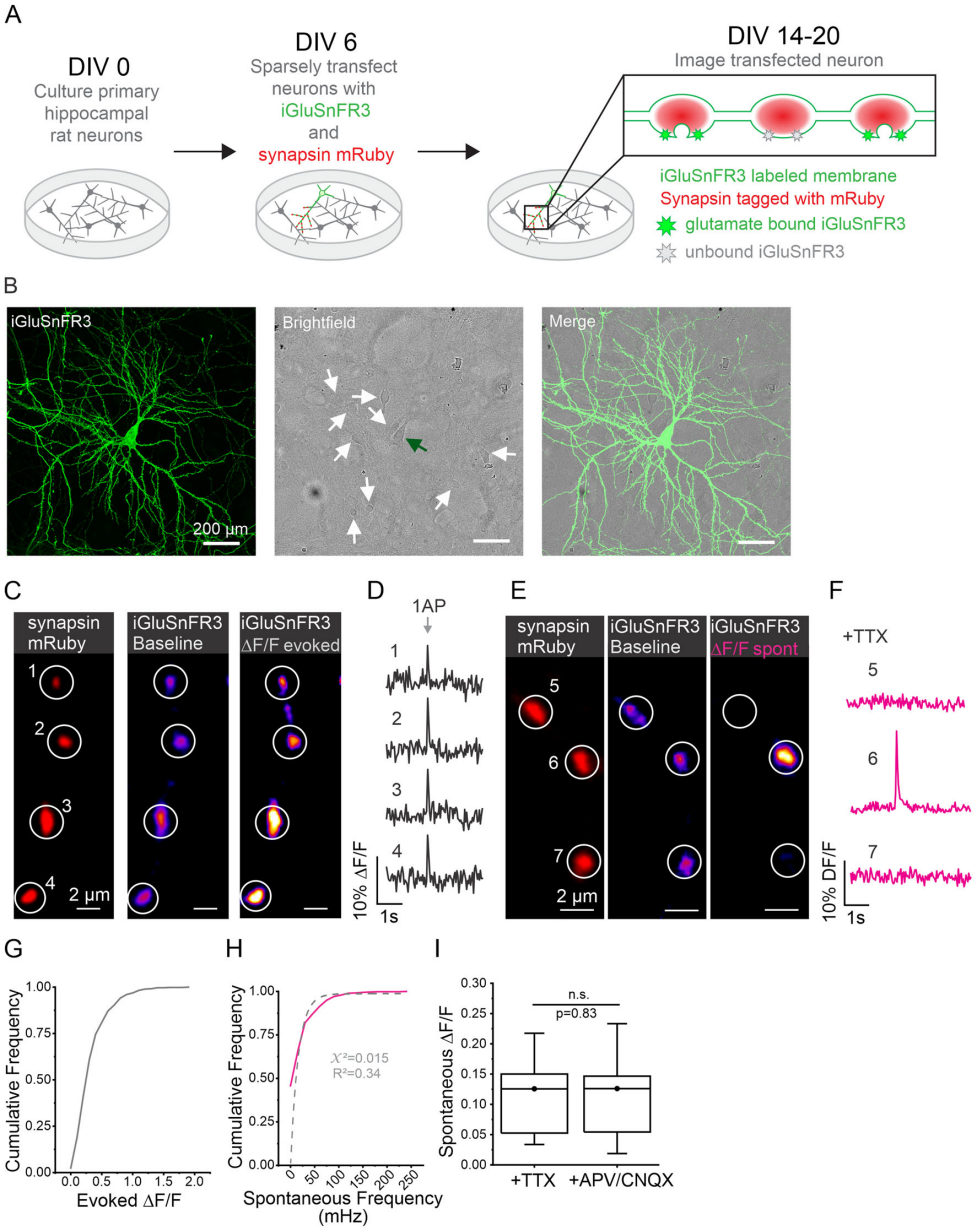
Evoked and spontaneous release events can be accurately detected and measured using iGluSnFR3. ***A***, Experimental protocol. On DIV 0, primary hippocampal neurons are harvested from newborn rat pups. On DIV 6, neurons are transfected with iGluSnFR3 and synapsin-mRuby plasmids. Imaging experiments are performed DIV 14–20 when neurons are expressing previously transfected plasmids. When glutamate is bound to iGluSnFR3, the indicator increases in fluorescence. ***B***, Representative images of sparsely transfected cell culture. The first panel shows fluorescent signal from iGluSnFR3-transfected cell fixed and tagged with GFP (see Methods). The second panel shows field of view through bright field with a green arrow indicating the cell body of transfected cell shown in the first panel and white arrows indicating surrounding cell bodies that have not been transfected. The third panel shows merging fluorescent and bright-field images. Note en passant boutons along axons. ***C***, Imaging evoked release events at individual boutons. mRuby-labeled synapsin is used to identify ROIs (1–4), and iGluSnFR3 signal is shown for axons at rest as well as the change in fluorescence exhibited during stimulation with a single AP shown as DF/F used to detect release events. ***D***, Corresponding traces for ROIs shown in C. DF/F traces showing evoked release events at individual boutons. ***E***, Imaging spontaneous release events at individual boutons. ROIs (5–7) are determined using synapsin-mRuby signal (same method as for evoked release shown in panel ***C***); iGluSnFR3 signal is measured only at boutons where spontaneous release event is occurring. ***F***, Corresponding traces for ROIs shown in ***E***. DF/F traces showing spontaneous release event are detected only at a single bouton (ROI 6) and not at adjacent boutons. ***G***, Cumulative frequency of magnitude of evoked events across individual boutons (*n* = 577 boutons from 13 cells). ***H***, Cumulative frequency of spontaneous release frequency at individual boutons represented by the magenta solid line, best fit of exponential function to data represented by gray dashed line (*n* = 577 boutons from 13 cells), *y* = *y*0 + A*(1−exp(−x/mu)); *y*0 = 0; *A* = 0.983; mu = 0.016. ***I***, Amplitude of spontaneous release events (DF/F) in the presence of TTX (*n* = 1,969 boutons from 21 cells) or APV/CNQX (*n* = 2,109 boutons from 24 cells), Mann–Whitney test, *p* = 0.83.

It is unclear how evoked and spontaneous release relates to one another within individual synaptic boutons. Assuming all boutons engage in spontaneous release at the same frequency, it is estimated that spontaneous release occurs at a rate between 0.01 and 0.03 Hz as measured using electrophysiological recordings of postsynaptic currents (Geppert et al., 1994; Murthy and Stevens, 1999). While electrophysiology provides excellent sensitivity and superior temporal resolution, these measurements are taken without knowledge of where spontaneous release events occur, a large knowledge gap given that a hippocampal neuron can have hundreds of individual synaptic connections. This lack of spatial resolution may be masking large amounts of variability in the balance of release types at individual synapses as has been suggested from measurements of presynaptic terminals of the NMJ (Choi et al., 2014; Astacio et al., 2022). On the other hand, prior assumptions hold that spontaneous release occurs stochastically at a homogenous frequency across all boutons (Rotshenker and Rahamimoff, 1970; Truckenbrodt and Rizzoli, 2014). Measuring both evoked and spontaneous release at the same individual presynaptic boutons would provide better understanding about if and how spontaneous release rates vary throughout a single axon. We measured both forms of release at boutons across the axons of individual cultured hippocampal neurons labeled with the fluorescent glutamate reporter iGluSnFR3, using synapsin-mRuby labeling to confirm measurements were only made at presynaptic boutons (Fig. 1*A*). In order to isolate single axons, we labeled less than 1% of neurons in a culture (Fig. 1*B*). Using field stimulation to generate an AP, we could detect evoked neurotransmitter release presynaptically at individual boutons along an axon (Fig. 1*C,D*). All stimulation was done in the presence of glutamate receptor blockers APV ((2*R*)-amino-5-phosphonovaleric acid) and CNQX (6-cyano-7-nitroquinoxaline-2,3-dione) to prevent background electrical activity. Independent of AP stimulation, flashes could be detected sporadically at individual mRuby-labeled boutons. Such flashes were of similar kinetics and magnitude to evoked release events, as seen in Figure 1*E,F*. Based on the frequency and magnitude of these events, we suspected that they were examples of spontaneous release. To confirm the nature of these events more thoroughly, we imaged axons in the presence of tetrodotoxin (TTX) for 2 min. Spontaneous release events were identified using a signal-to-noise analysis as previously described (Wang et al., 2022) as well as here (see Methods). We found that spontaneous release occurred at an average frequency of 0.021 ± 0.035 Hz or 21 mHz (2014 boutons in 45 cells) per bouton and that both the magnitude of evoked release (Fig. 1*G*) and frequency of spontaneous release (magenta line; Fig. 1*H*) were quite heterogeneous. When quantifying the frequency of spontaneous release, we noted that it was not well fit by a single exponential (dashed line; Fig. 1*H*). When we compared the amplitudes of spontaneous release events detected in the presence of TTX with those recorded in the presence of glutamate blockers APV and CNQX without stimulation, they were indistinguishable (Fig. 1*I*). These results demonstrate that we can accurately detect both evoked and spontaneous release events using iGluSnFR3 and that our sampling frequency is adequate to resolve the most, if not all, spontaneous fusion events. Given the sensitivity of our optical detection method, we were unsurprised to observe an average frequency of spontaneous release events similar to what has been reported using electrophysiology. Taken together, this suggests that our detection method can accurately report spontaneous and evoked release events, allowing us to investigate a potential relationship between these two forms of neurotransmitter release at single presynaptic boutons.

### Spontaneous release frequency and evoked release magnitude are positively correlated

**Figure 2.**
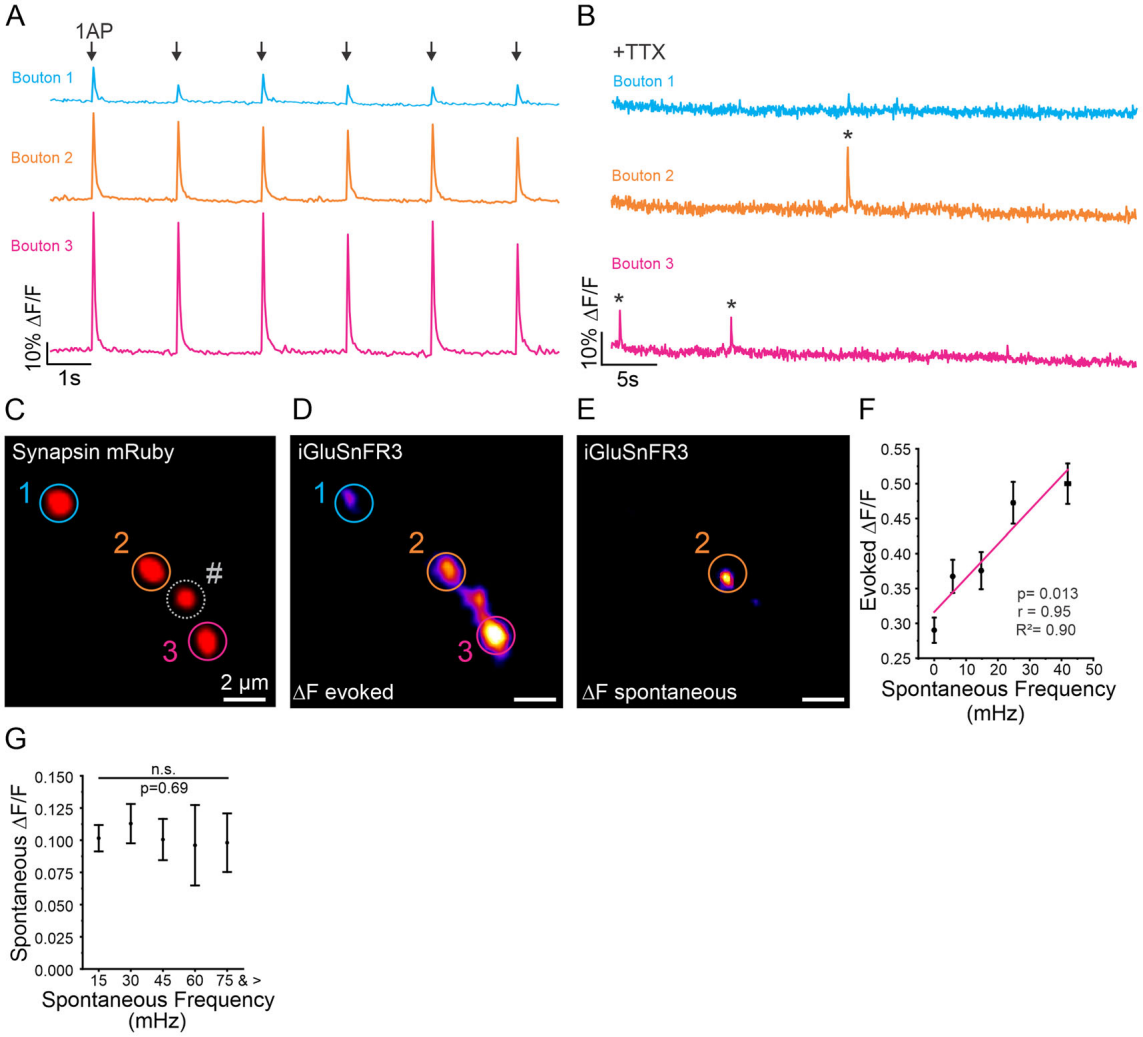
Evoked release amplitude is correlated with spontaneous release frequency at individual boutons. ***A***, Representative traces of iGluSnFR3 signal from three individual boutons along the same axon (corresponding to ROIs 1–3 in Fig. 1*C*) in response to six single AP stimulations. ***B***, Representative traces of iGluSnFR3 signal from the same boutons shown in (***A***) recording spontaneous release events in the presence of TTX. ***C***, Representative image of synapsin-mRuby and bouton selection (see Methods). # indicates bouton that was analyzed; however, individual traces are not shown here. ***D***, Representative images of iGluSnFR3 change in fluorescence (DF/F) in response to single AP stimulation at three individual boutons. ***E***, Representative image of iGluSnFR3 (DF/F) during a spontaneous release event. ***F***, Relationship between spontaneous release frequency and evoked release magnitude. Note that the linear regression analysis (magenta line) relationship between the frequency of spontaneous release events and magnitude of evoked release at individual boutons (binned in groups of 50) shows strong positive correlation (*n* = 577 boutons from 13 cells), *r* = 0.95, *R*^2^ = 0.90, *p* = 0.013. ***G***, Comparison of spontaneous release magnitude compared to spontaneous release frequency at individual boutons (*n* = 577 boutons, from 13 cells), Kruskal–Wallis ANOVA, *p* = 0.69, *χ*^2^ = 0.21. * Indicates spontaneous release event.

While the mechanisms regulating heterogeneity in *Pr* across boutons during evoked release have been explored, varying rates of spontaneous release across individual presynaptic boutons is much less well understood. In some cases, the two forms of release are thought to be antagonistic when postsynaptically measuring both forms of release in AZs of the NMJ (Melom et al., 2013). To further investigate this, we sought to determine how spontaneous release frequency correlated with evoked release magnitude at individual presynaptic boutons. We measured spontaneous release frequency in the presence of APV and CNQX for 2 min and then stimulated neurons with 20 APs at 0.5 Hz, which reliably caused neurotransmitter release in most stimulations to measure individual evoked release events (Fig. 2*A,B*). Boutons with larger evoked release amplitudes often had greater spontaneous release frequencies (Fig. 2*C–E*). Indeed, when we ran a linear regression comparing evoked amplitude to spontaneous release frequency, we found the two to be positively correlated (*R*^2^ = 0.90, *p* < 0.05) (Fig. 2*F*). To ensure that variations in iGluSnFR3 expression were not contributing to higher detection rates at some boutons, we compared the spontaneous release amplitude to the spontaneous release frequency. Given that a spontaneous release event is widely accepted as the release of a single SV (Fatt and Katz, 1952; Frerking et al., 1997), we expected the amplitudes of spontaneous release events to be similar across boutons. We found no significant difference (Fig. 2*G*), indicating that the positive correlation between evoked release magnitude and spontaneous release frequency is not an artifact of iGluSnFR3 expression related to signal noise detection. These results indicate that boutons with a higher probability for evoked release (P*r*) also have a high propensity for spontaneous release, suggesting that factors contributing to evoked release may also be involved in spontaneous release.

### Spontaneous release frequency is predicted by RRP size

**Figure 3.**
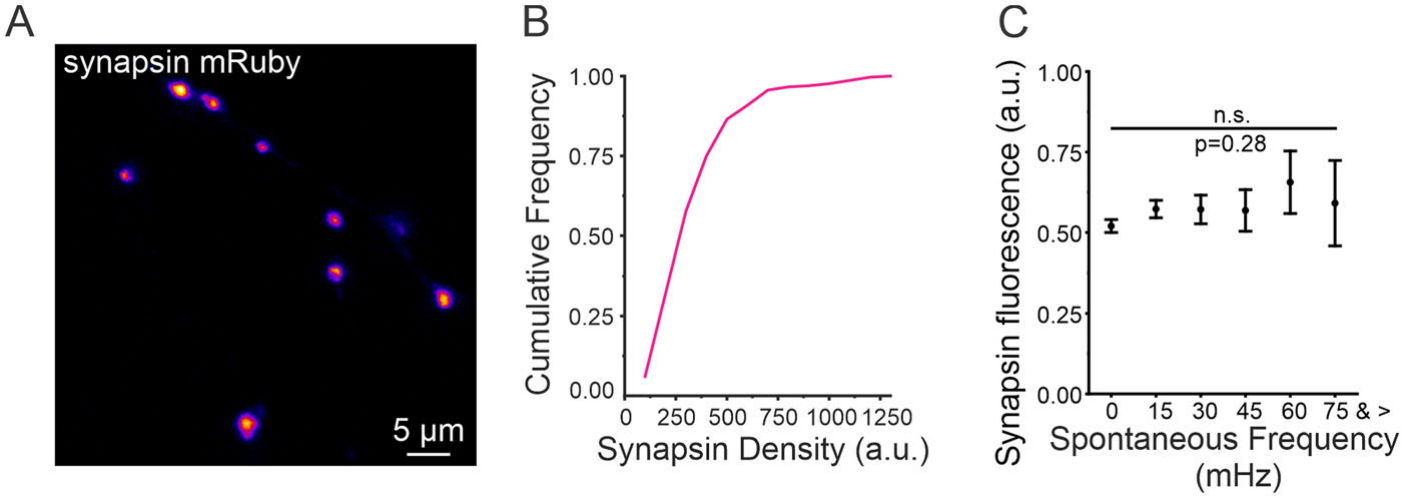
Synapsin intensity is not correlated with spontaneous release frequency. ***A***, Representative image of synapsin-mRuby signal in individual boutons along the axon of a hippocampal neuron. ***B***, Cumulative frequency of synapsin-mRuby signal (*n* = 296 boutons from 7 cells). ***C***, Synapsin fluorescence (normalized to max of each cell) compared to spontaneous release frequency (*n* = 296 boutons from 7 cells), Kruskal–Wallis ANOVA, *p* = 0.28, *χ*^2^ = 16.00.

We next sought to determine if the correlation between evoked release magnitude and spontaneous release frequency could be attributed to a higher number of vesicles at some boutons. It is well established that synapsin is closely associated with synaptic vesicles (SVs) within presynaptic boutons (Benfenati et al., 1992; Rosahl et al., 1995; Clementi et al., 1999). We used an mRuby-labeled synapsin to estimate the relative quantity of SVs within individual boutons (Fig. 3*A*). As suggested in the literature (Chi et al., 2003), we found the synapsin intensity in each individual bouton to be variable which we normalized within each neuron to control for overall differences in expression (Fig. 3*B*). When we compared the normalized intensities of synapsin, we found that the relative amount of synapsin at individual boutons was not correlated with the rate of spontaneous release (Fig. 3*C*). These data suggest that spontaneous release is not a function of total vesicle quantity within individual boutons.

**Figure 4.**
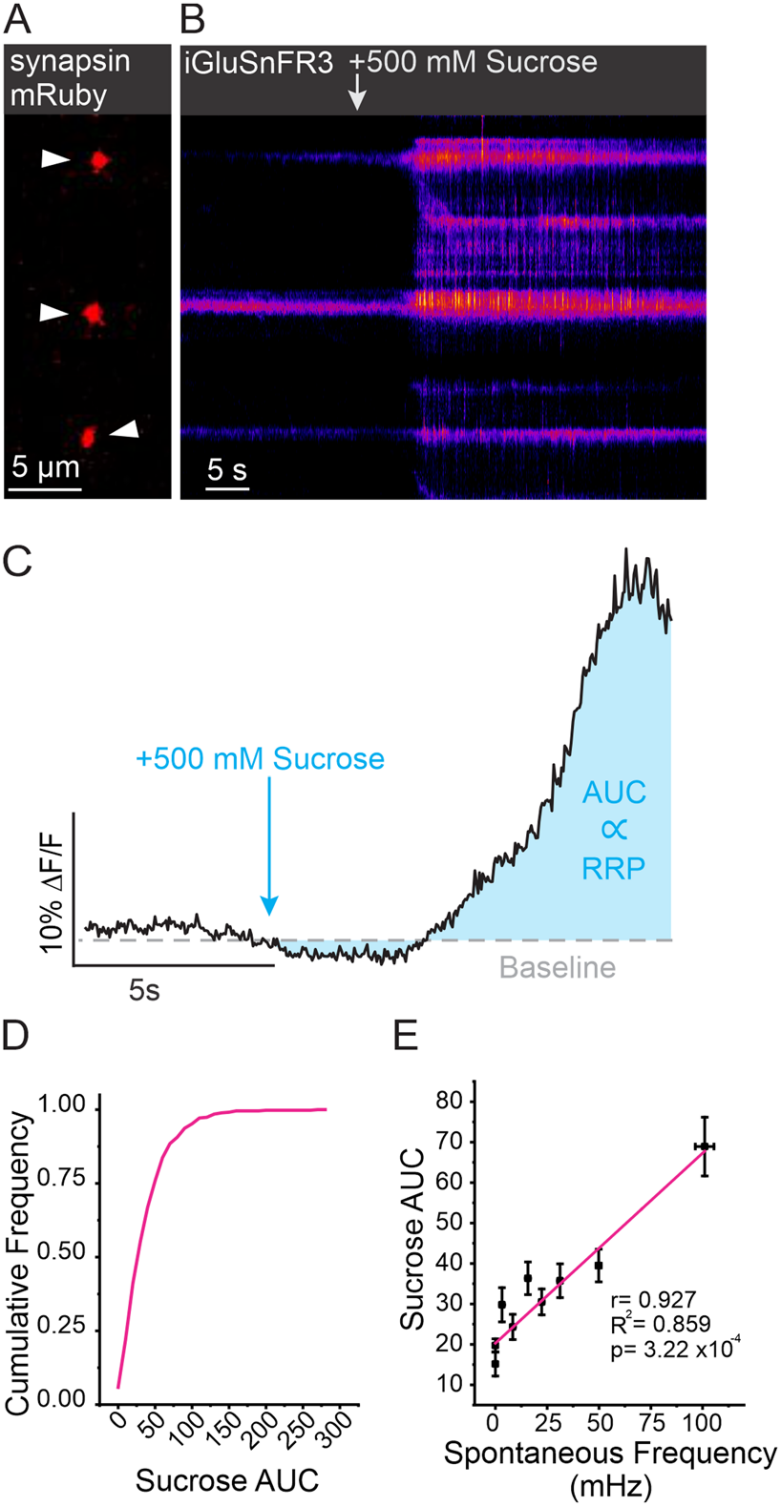
RRP size as measured by sucrose application is correlated with spontaneous release frequency. ***A***, Example ROIs identified using synapsin-mRuby, indicated by arrowheads. ***B***, Corresponding iGluSnFR3 kymograph from boutons in ***A*** with arrow indicating sucrose application. ***C***, Example trace with schematic depicting measurements of RRP using sucrose. Integration of the fluorescent signal (AUC) of iGluSnFR3 DF/F signal for 10 s after sucrose was applied is used to approximate RRP size. ***D***, Cumulative frequency of AUC of iGluSnFR3 signal in response to sucrose across individual boutons (*n* = 460 boutons from 8 cells). ***E***, Linear regression between AUC of sucrose response and spontaneous release frequency at individual boutons showing highly positive correlation (*n* = 460 boutons from 9 cells), binned in groups of 50, *r* = 0.927, *R*^2^ = 0.859, *p* = 3.22 × 10^−4^.

**Figure 5.**
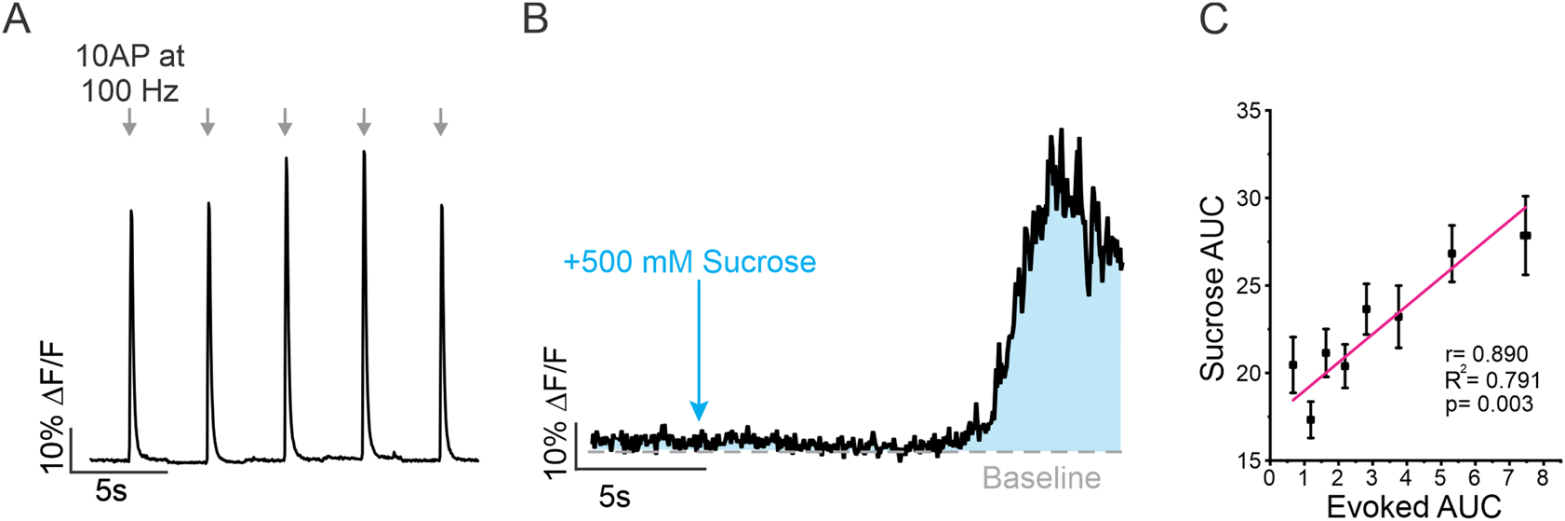
Response to high-frequency stimulation is positively correlated with response to sucrose. ***A***, Example trace depicting a single bouton DF/F iGluSnFR3 response to five individual 10 APs at 100 Hz stimulations. ***B***, Example trace of iGluSnFR3 DF/F signal in response to sucrose application from the same bouton as trace in Figure 5*A*. ***C***, Linear regression between AUC of sucrose response and AUC of high-frequency stimulation at individual boutons showing highly positive correlation (*n* = 447 boutons from 9 cells), binned in groups of 50, *r* = 0.890, *R*^2^ = 0.791, *p* = 0.003.

Within individual boutons, SVs are organized into functional pools, with only a fraction participating in release (Denker and Rizzoli, 2010; Alabi and Tsien, 2012). The RRP is a pool of vesicles that are docked and primed, meaning they are closely associated with the plasma membrane and ready for immediate release upon the arrival of an AP (Kaeser and Regehr, 2017). To determine if spontaneous release frequency was correlated with RRP size, we took advantage of a classic physiological technique by applying a hypertonic (500 mOsm) sucrose solution to trigger fusion of vesicles in the RRP (Fatt and Katz, 1952; Rosenmund and Stevens, 1996; Schotten et al., 2015). This is possible, thanks to the unique rapid on-neuron kinetics and high *K*_fast_ of iGluSnFR3 that make iGluSnFr3 sensitive to detecting multifusion events without saturation (Aggarwal et al., 2023). At boutons labeled with synapsin-mRuby, a dramatic change in iGluSnFR3 fluorescence was observed within 5 s of sucrose application (Fig. 4*A,B*). As a relative measurement of RRP size at individual boutons, we calculated the area under the curve (AUC) of iGluSnFR3 signal for 10 s following the application of sucrose (Fig. 4*C*). We found that boutons, unsurprisingly, had a large distribution of RRP sizes (Fig. 4*D*). However, when compared using a linear regression, we found that spontaneous release frequency was highly correlated with RRP size (*R*^2^ = 0.86, *p* < 0.001; Fig. 4*E*). In an additional set of experiments, we further evaluated our method of RRP measurement by comparing individual boutons response to a high-frequency train of 10 APs delivered at 100 Hz which served to rapidly exhaust the RRP before extensive refilling from other vesicle pools (Fig. 5*A*). This measurement was then compared in the same synapses to the magnitude of a hypertonic sucrose response (Fig. 5*B*). We compared the AUC for both types of response using a linear regression and found that at individual boutons, although the response to each stimulation had quite different kinetics, the high-frequency stimulation was positively and significantly correlated with the response to sucrose application suggesting agreement between the two measurements (*R*^2^ = 0.791, *p* = 0.003; Fig. 5*C*). Moreover, these results demonstrate that spontaneous release frequency is positively correlated with the number of vesicles in the RRP of an individual bouton.

### Acute changes in RRP size dramatically alter the rate of spontaneous neurotransmission

**Figure 6.**
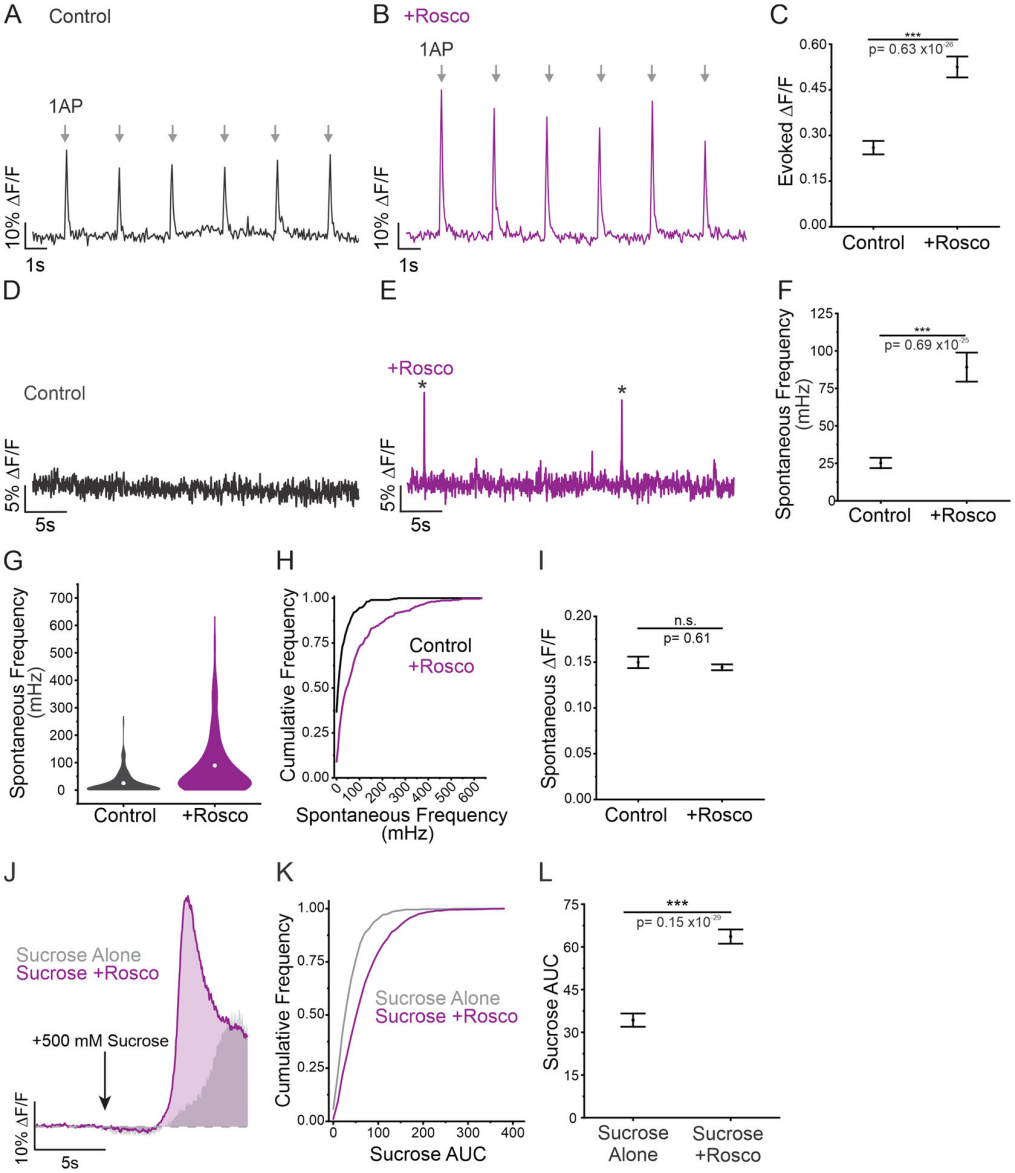
Increase in RRP size is correlated with increase in spontaneous release frequency. ***A,B***, Representative DF/F traces of six evoked release events, each in response to a 1 AP stimulation (arrows), before and after roscovitine treatment at the same single bouton. ***C***, Magnitude of evoked release in control conditions or in roscovitine treatment (*n* = 299 boutons from 7 cells), Mann–Whitney test, *p* < 0.001. ***D,E***, Representative DF/F traces of spontaneous release events before and after roscovitine treatment at the same single bouton. * Indicates spontaneous release event. ***F***, Spontaneous release frequency in control conditions compared to roscovitine treatment, *n* = 299 boutons from seven cells, Mann–Whitney test, *p* < 0.001. ***G***, Volcano plots showing the spread of spontaneous release frequencies before and after roscovitine treatment. ***H***, Cumulative frequency of spontaneous release frequency across individual boutons in control conditions and roscovitine treatment. ***I***, Amplitude of spontaneous release events in control conditions and in roscovitine treatment, *n* = 299 boutons from seven cells, Mann–Whitney test, *p* = 0.61. ***J***, Unpaired example traces depicting the response to sucrose alone (gray) and the response to sucrose + roscovitine treatment (purple). Gray shading is showing data from controls (sucrose alone) for comparison and is taken in Figure 4*C*. ***K***, Cumulative frequency of sucrose AUC sizes in response to sucrose alone or sucrose + roscovitine. ***L***, AUC in response to sucrose alone or sucrose + roscovitine treatment, sucrose alone: *n* = 460 boutons from nine cells; sucrose + roscovitine: *n* = 460 boutons from seven cells, Mann–Whitney test, *p* < 0.001. For all statistics, exact *p* values are given in figure panels.

**Figure 7.**
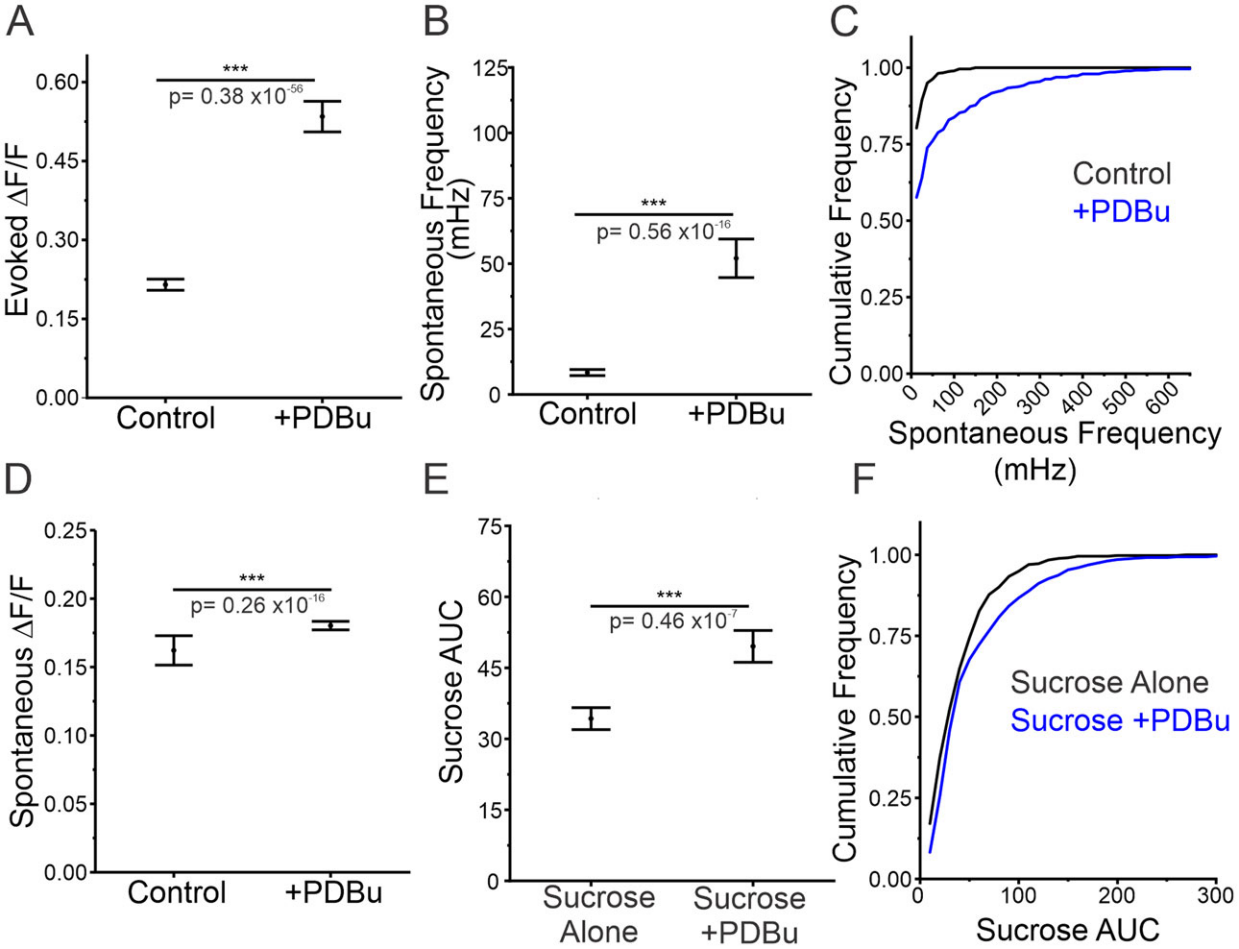
Increasing RRP size using PDBu results in increase in spontaneous release frequency. ***A***, Magnitude of evoked release in control conditions or in PDBu treatment (*n* = 481 boutons from 7 cells), Mann–Whitney test, *p* = 0.38 × 10^−56^ < 0.001. ***B***, Spontaneous release frequency in control conditions or in PDBu treatment, *n* = 481 boutons from seven cells, Mann–Whitney test, *p* < 0.001. ***C***, Cumulative frequency of spontaneous release frequency across individual boutons in control conditions and PDBu treatment. ***D***, Amplitude of spontaneous release events in control conditions or in PDBu treatment, *n* = 481 boutons from seven cells, Mann–Whitney test, *p* = 0.26 × 10^−16^ < 0.001. ***E***, AUC in response to sucrose alone or sucrose + PDBu, sucrose alone: *n* = 460 boutons from nine cells; sucrose + PDBu: *n* = 481 boutons from seven cells, Mann–Whitney test, *p*^7^ < 0.001. ***F***, Cumulative frequency of sucrose AUC sizes in response to sucrose alone or sucrose + PDBu. For all statistics, exact *p* values are given in figure panels.

Given the correlation between spontaneous release frequency and RRP size, we next sought to determine if spontaneous release frequency is altered in response to acute changes in RRP size. Since genetic approaches would not allow us to rapidly manipulate RRP size, we opted for a pharmacological approach. We utilized roscovitine, which has been shown to increase the releasable pool and RRP size through inhibition of CDK5 (Kim and Ryan, 2010). Acute application of roscovitine resulted in a significant increase in evoked amplitude within 10 min of application (Fig. 6*A–C*). In support of our previous findings, spontaneous release frequency significantly increased in response to roscovitine treatment (Fig. 6*D–F*). Additionally, we observed an increase in the proportion of boutons engaging in spontaneous release with 90% of boutons demonstrating spontaneous release following roscovitine application compared to 63% in control conditions (Fig. 6*G,H*). While the detection of spontaneous release could be enhanced by changes in amplitude, we did not see any statistically significant changes in spontaneous release amplitude from treatment with roscovitine (Fig. 6*I*), suggesting that the treatment does not interfere with iGluSnFR3 function. We used the same sucrose application paradigm as previously shown (Fig. 6*C*) to confirm the increase in RRP size (Fig. 6*J*). We observed a positive shift in the distribution of RRP sizes, as well as a significant increase in RRP size following treatment with roscovitine (Fig. 6*K,L*). Our results align with previously published data suggesting that roscovitine treatment increases *Pr* by increasing RRP size (Kim and Ryan, 2010; Marra et al., 2012). It was recently shown that phorbol 12,13-dibutyrate (PDBu) treatment can also acutely increase RRP size and in turn increase spontaneous release frequency (Feldthouse et al., 2023). We repeated our measurements using PDBu as an alternative to roscovitine mediated increases in RRP size. Acute application of PDBu increased evoked release threefold (Fig. 7*A*) while also enhancing the frequency of spontaneous release fourfold as well as a small increase (11%) in the amplitude of spontaneous release (Fig. 7*B–D*). This also resulted in an increase in the size of the RRP from PDBu application (45% increase) as also seen by application of roscovitine (Fig. 7*E,F*). Thus, two separate manipulations that enhance RRP size also predictably enhance spontaneous release frequency at the single bouton level. Thus, the frequency of spontaneous release can quickly mirror dynamic changes in RRP size caused by roscovitine or PDBu.

### Acute changes in Pv are unrelated to the rate of spontaneous neurotransmission

**Figure 8.**
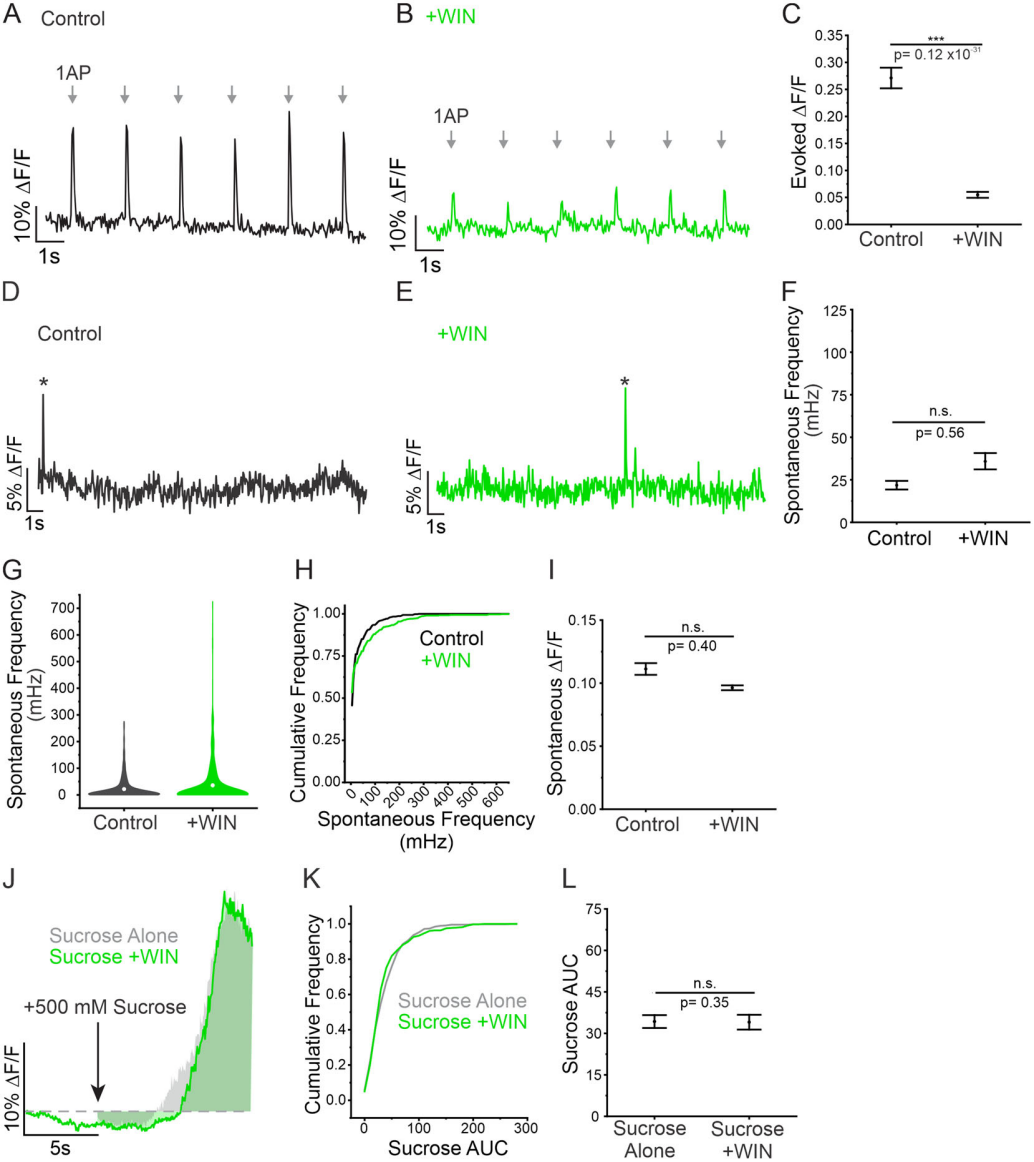
Changes to evoked release independent of changes to RRP size do not affect spontaneous release frequency. ***A,B***, Representative DF/F traces of six evoked release events, each in response to a 1 AP stimulation (arrows), before and after WIN treatment at the same single bouton. ***C***, Magnitude of evoked release in control conditions or in WIN treatment, *n* = 615 boutons from 10 cells, Mann–Whitney test, *p* < 0.001. ***D,E***, Representative DF/F traces of spontaneous release events before and after WIN treatment at the same single bouton. * Indicates spontaneous release event. ***F***, Spontaneous release frequency in control conditions compared to WIN treatment, *n* = 615 boutons from 10 cells, Mann–Whitney test, *p* = 0.56. ***G***, Volcano plots showing the spread of spontaneous release frequencies before and after WIN treatment. ***H***, Cumulative frequency of spontaneous release frequency across individual boutons in control conditions and in WIN treatment. ***I***, Amplitude of spontaneous release events in control conditions and in WIN treatment, *n* = 615 boutons from 10 cells, Mann–Whitney test, *p* = 0.40. ***J***, Unpaired example traces depicting the response to sucrose alone (gray) and the response to sucrose + WIN treatment (green). ***K***, Cumulative frequency of sucrose AUC sizes in response to sucrose alone or sucrose + WIN. ***L***, AUC in response to sucrose alone or sucrose + WIN, sucrose alone: *n* = 460 boutons from nine cells; gray shading is showing data from controls (sucrose alone) for comparison and is taken in Figure 4*C*; sucrose + WIN: *n* = 435 boutons from six cells, Mann–Whitney test, *p* = 0.35. For all statistics, exact *p* values are given in figure panels.

It is well established that RRP size influences evoked release (Ariel and Ryan, 2010; Ariel et al., 2013; Kaeser and Regehr, 2017), and thus far, our results demonstrate a strong correlation between spontaneous release frequency and evoked release magnitude. The probability of an AP opening VGCCs strongly contributes to Pv, the second variable affecting evoked release. For this reason, we sought to alter evoked release magnitude without affecting RRP to determine if changes in Pv would influence spontaneous release frequency. Pv was manipulated by the CB1 receptor agonist WIN 55,212-2 mesylate salt (WIN), which acts by decreasing the opening of VGCCs during AP stimulation (Szabó et al., 2014). Indeed, we found that acute application of WIN significantly impaired evoked release (Fig. 8*A–C*). Conversely, spontaneous release was not affected by these acute CB1-mediated decreases in Pv (Fig. 8*D–F*). Following WIN treatment, the frequency and amplitude of spontaneous release were not significantly altered, and the distribution of release frequencies closely mirrored that of control conditions (Fig. 8*G–I*). To ensure that WIN was only affecting Pv, and not altering RRP size, we measured the response to sucrose using the same method as previously shown (Fig. 4*C*) and found that RRP size was not significantly decreased by WIN (Fig. 8*J–L*). These results indicate that spontaneous release frequency is not affected by changes in Pv, when RRP is left intact. This supports spontaneous release as an independently regulated form of neurotransmission that is not influenced by the same rules as evoked release. Instead, both spontaneous and evoked release depend on a common physiological variable: the quantity of docked vesicles that occupy the RRP.

### Presynaptic calcium indicators are not sensitive enough to predict or identify spontaneous release

**Figure 9.**
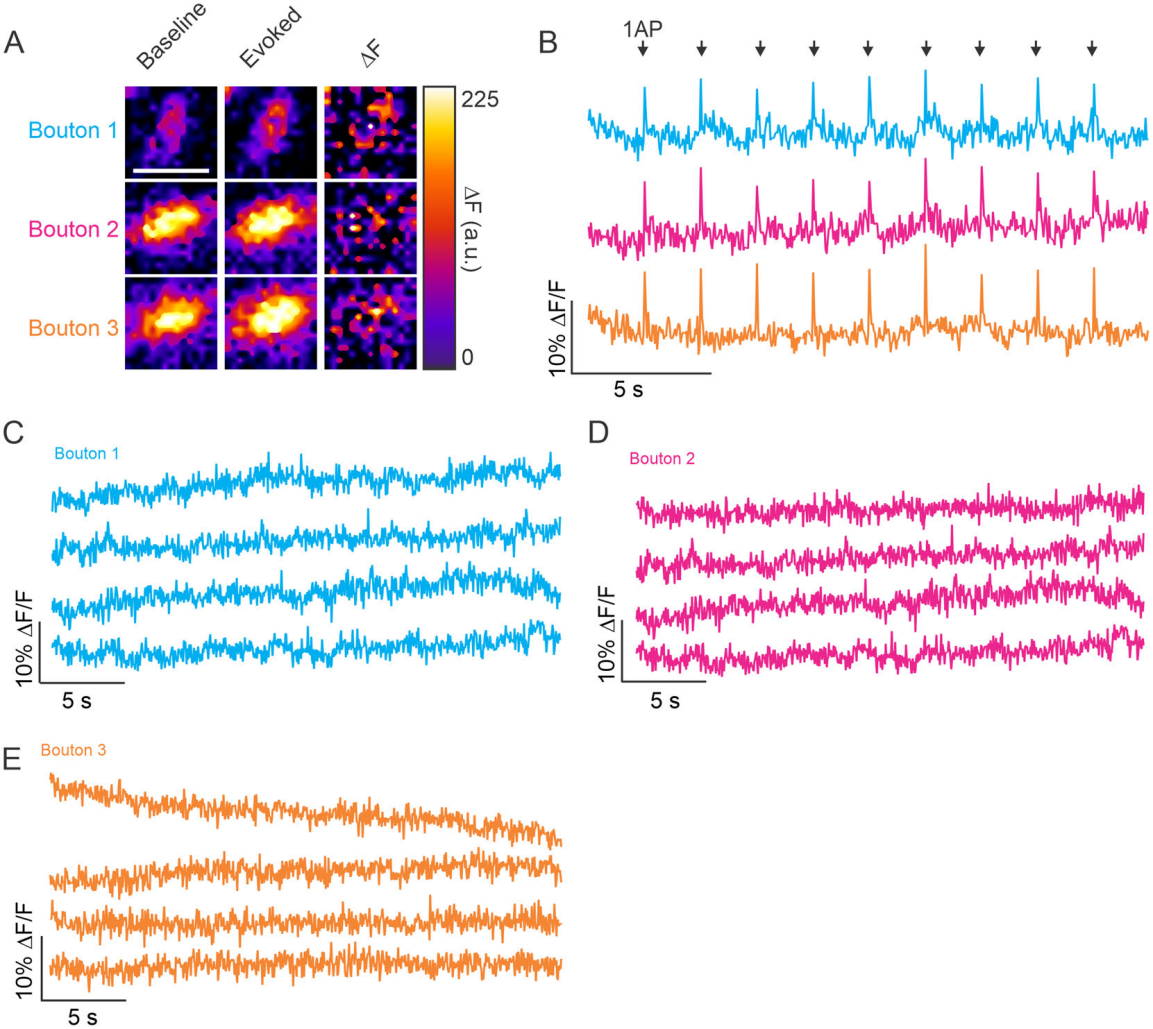
Presynaptic measurements of calcium using jGCaMP8f fail to detect calcium transients at frequencies representing spontaneous release. ***A***, Baseline, evoked, and DF of jGCaMP8f fluorescent signal are shown for three individual boutons, scale bar = 2 mm. ***B***, Traces demonstrating the jGCaMP8f DF/F in response to nine single AP stimulations across three individual boutons located along the same axon. ***C***, Example traces from bouton 1 (Fig. 9*B*) of jGCaMP8f in the absence of stimulation. ***D***, Example traces from bouton 2 (Fig. 9*B*) of jGCaMP8f in the absence of stimulation. ***E***, Example traces from bouton 3 (Fig. 9*B*) of jGCaMP8f in the absence of stimulation.

**Figure 10.**
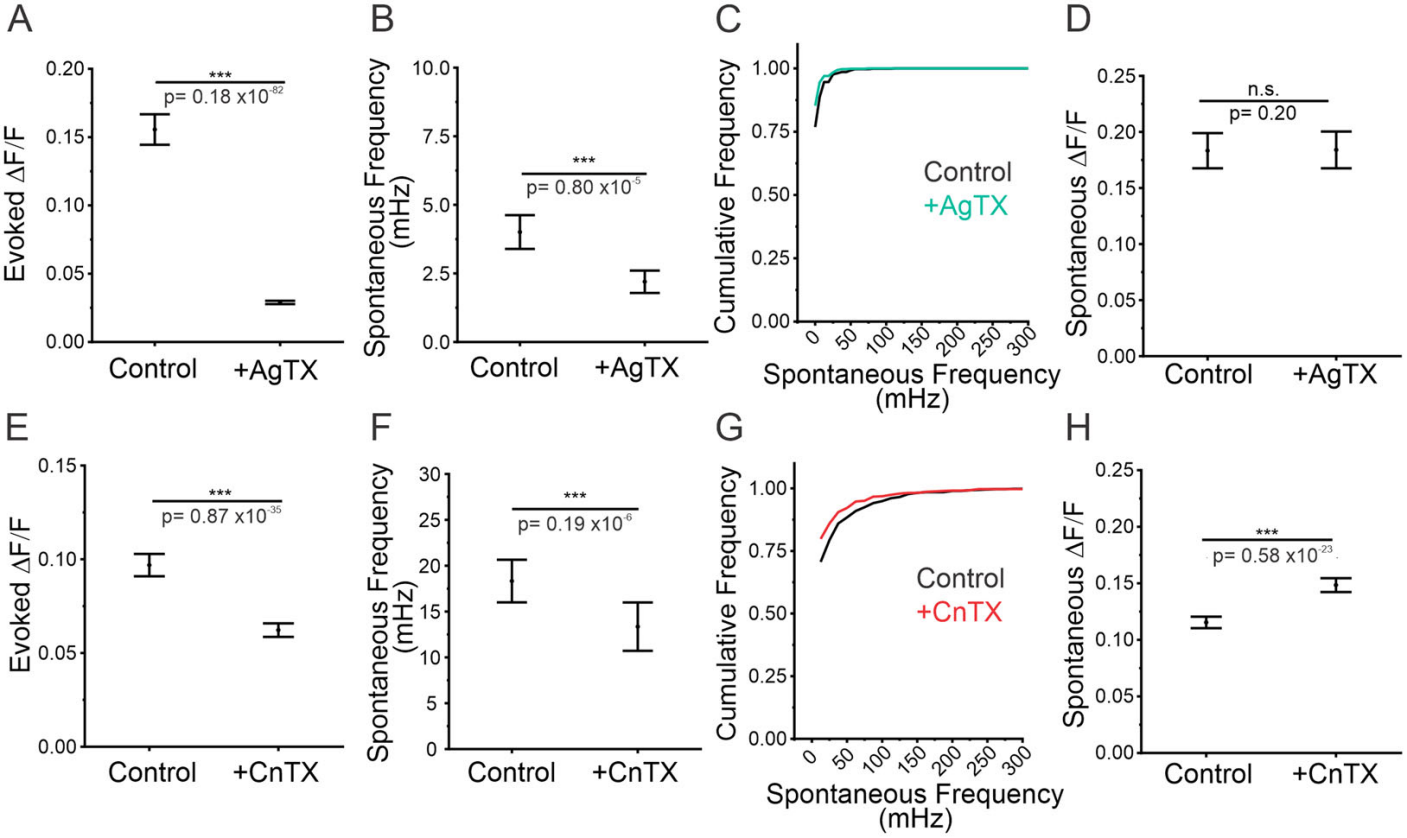
Blocking P/Q- and N-type VGCCs partially reduces the frequency of spontaneous release. ***A***, Magnitude of evoked release in control conditions or in AgTX treatment, *n* = 630 boutons from seven cells, Mann–Whitney test, *p* < 0.001. ***B***, Spontaneous release frequency in control conditions or in AgTX treatment, Mann–Whitney test, *p* = 0.80 × 10^−5^ < 0.001. ***C***, Cumulative frequency of spontaneous release frequency across individual boutons in control conditions and in AgTX treatment. ***D***, Amplitude of spontaneous release events in control conditions or in AgTX treatment, Mann–Whitney test, *p* = 0.20. ***E***, Magnitude of evoked release in control conditions or in CnTX treatment, *n* = 664 boutons from nine cells, Mann–Whitney test, *p* = 0.87 × 10^−35^ < 0.001. ***F***, Spontaneous release frequency in control conditions or in CnTX treatment, Mann–Whitney test, *p* = 0.19 × 10^−6^ < 0.001. ***G***, Cumulative frequency of spontaneous release frequency across individual boutons in control conditions and in CnTX treatment. ***H***, Amplitude of spontaneous release events in control conditions and in CnTX treatment, Mann–Whitney test, *p* < 0.001. For all statistics, exact *p* values are given in figure panels.

Our results using WIN to impair the opening of voltage-gated calcium channels through the Gi metabotropic pathway selectively impaired evoked, but spared spontaneous, release. Although the mechanisms for initiating spontaneous release are unknown, changing extracellular calcium, as well as pore-blocking of voltage-gated calcium channels, can significantly alter the frequency of spontaneous release (Ermolyuk et al., 2013; Tsintsadze et al., 2017; Babiec and O’Dell, 2018). This suggests that the influx of calcium signals independent of APs could initiate spontaneous release likely through calcium channels on the plasma membrane or endoplasmic reticulum. We expressed jGCaMP8F (Zhang et al., 2023), a highly sensitive indicator of calcium, to try and detect calcium influx during evoked stimulation of single APs in the same conditions we used for measuring glutamate release. We could detect a robust signal when delivering an AP at virtually all labeled boutons (Fig. 9*A,B*). However, we could not detect any calcium signals that were of similar magnitude occurring spontaneously, or any signal above background at individual boutons (examples shown in Fig. 9*C–E*). However, utilizing GluSnFR3's sensitivity, we measured the contribution of voltage-gated P/Q- (Ca_V_2.1) and N-type (Ca_V_2.2) channels to evoked and spontaneous release. We found that blocking P/Q-type channels reduced the magnitude of evoked release by 80% while also reducing the frequency of spontaneous release by 45%, yet leaving the amplitude of spontaneous release events insignificantly changed in Figure 10*A–D*. Additionally, we found similar contributions of N-type channels to evoked release (35% decrease) while also reducing the frequency of spontaneous release by 27% as well as increasing the magnitude of spontaneous events (28%) as seen in Figure 10*E–H*. These results agree with prior measurements of spontaneous release frequency using electrophysiology, suggesting a partial contribution from voltage-gated calcium channels (Ermolyuk et al., 2013), although not all cell types share this phenotype (Tsintsadze et al., 2017). Ultimately, these results suggest at least a partial reliance of spontaneous release on calcium influx at the plasma membrane, but either the magnitude or kinetics of this signal is much different than that from an AP and thus, can evade presynaptic detection using jGCaMP8f, the most sensitive protein-based fluorescent calcium indicator to date (Zhang et al., 2023).

## Discussion

In this study, we used an optical sensor of glutamate release, iGluSnFR3, to detect both evoked and spontaneous neurotransmission, as well as to quantify the RRP at the level of individual hippocampal boutons. We report a correlation between the magnitude of evoked release and the frequency of spontaneous release at the level of individual boutons (Fig. 2). Evoked release comprises two main physiological factors: the number of docked SVs that make up the RRP and the probability of vesicle release (Pv). It has been nearly impossible to measure these two factors separately in individual boutons because existing methods are not sensitive enough to finely resolve spontaneous release both spatially and temporally. We took advantage of the much larger dynamic range and lower saturation of iGluSnFR3 (Aggarwal et al., 2023) compared to original variants of this indicator (Marvin et al., 2013), to make quantitative measurements of the RRP at individual boutons (Fig. 4). While the magnitude of evoked release was correlated with the rate of spontaneous release, we found that the rate of spontaneous release was almost perfectly predicted by RRP size at the level of individual boutons. Moreover, dynamic increases in RRP size in response to acute treatments of a CDK5 inhibitor, roscovitine, result in corresponding increases in spontaneous release frequency (Fig. 6). Taken together, these findings demonstrate an exciting connection between evoked release and spontaneous release. Based on our results, the RRP appears to be central to both spontaneous and evoked release. However, the other central feature of evoked transmission (*Pr*) is Pv. We found that there is no connection between Pv and spontaneous release, suggesting that spontaneous release occurs independently of the other central modulator of evoked release. We found that acute changes to Pv, such as the inhibition of VGCCs during evoked release by activation of cannabinoid receptors, only altered evoked transmission and not spontaneous. Therefore, we conclude that there is no connection between spontaneous and evoked release other than a shared dependence on the RRP. This finding does not rule out a contribution of VGCCs for initiating spontaneous release, and our recordings found that both P/Q- and N-type channels have at least a partial role in spontaneous release (Fig. 10). Overall, these results strongly suggest that the frequency of spontaneous release is a direct result of a fundamental synaptic property, RRP size.

### Pv and RRP as properties of evoked release to help understand relationship with spontaneous release

The earliest electrophysiological measurements of spontaneous release suggested it was a purely stochastic phenomenon, but without really considering aspects of Pr such as Pv and RRP (Rotshenker and Rahamimoff, 1970). Attempts to measure vesicle fusion types with styryl dyes suggested a synergistic relationship between evoked and spontaneous release (Prange and Murphy, 1999). This work relied on loading endocytosed compartments where dyes could mix during endosome formation and was not able to dissect vesicle pools in fine detail. More dynamic measurement of individual fusion events using calcium in the neuromuscular junction (NMJ) of *Drosophila* followed, which came to an opposite conclusion and suggested an antagonistic relationship between the two forms of release and advocated for specialization at release cites (Melom et al., 2013; Peled et al., 2014). However, more recent work at the NMJ of *Drosophila* found a more synergistic relationship between evoked amplitudes and frequency of spontaneous fusion at mature synapses, when measured only at mature release sites as marked by enrichment of the *Drosophila*-specific AZ protein Bruchpilot (Grasskamp et al., 2023). Evidence supporting a synergistic relationship between spontaneous release and evoked release at the NMJ was identified in zebrafish using sophisticated tracking methods of individual vesicle recycling during both types of fusion (Egashira et al., 2022). Outside of the NMJ, postsynaptic measurements of evoked and spontaneous release using an older higher-affinity form of a glutamate sensor (iGluSnFR) did not see a relationship between Pv and spontaneous release (Farsi et al., 2021; Wang et al., 2022), but did in one case; note a correlation between evoked amplitude and spontaneous release suggesting a potential role for the RRP (Farsi et al., 2021). However, the original iGluSnFR sensor is easily saturated and cannot be used to make a direct measurement of the RRP. Here, thanks to the development of a glutamate sensor with a much higher dynamic range (iGluSnFR3; Aggarwal et al., 2023), it was possible to make functional measurements of RRP size presynaptically, as well as measure spontaneous release frequency and evoked amplitudes at the individual boutons of hippocampal neurons. Excitingly, we found a very strong correlation between spontaneous frequency and RRP size as measured by sucrose (Fig. 4), which was also temporally linked by dynamically modulating the RRP size at individual synapses (Figs. 6, 7).

### The RRP is a dynamic pool that provides vesicles for both spontaneous and evoked release

RRP size can vary dramatically across boutons from several types of neurons in the CNS (Ariel et al., 2013; Jung et al., 2021; Chipman et al., 2022). In hippocampal neurons taken from brain slices, the size of the AZ can vary by more than an order of magnitude up to 0.2 mm^2^, with the number of docked vesicles varying between 0 and 20 with an average of 6 (Chipman et al., 2022). While the differences in spontaneous release that we observe are not stochastic at the level of individual boutons assuming identical rates of spontaneous fusion, they could still be a stochastic property of all docked vesicles, accounting for observed variations in spontaneous release frequency. Studies have shown that spontaneous vesicle fusion is controlled by separate molecular classes of proteins (Geppert et al., 1994; Huntwork and Littleton, 2007; Khan and Regehr, 2020) and is regulated in different nanolocations within the AZ (Guzikowski and Kavalali, 2021; Wang et al., 2022). This could imply that SVs in the RRP are split into separate molecular classes, designated to either evoked or spontaneous release. However, our results demonstrate that docked vesicles that fill the RRP are responsible for both evoked and spontaneous neurotransmission. It may appear that these two observations are antagonistic; however, that would only be the case if vesicle docking and priming were linear and nondynamic events, i.e., that a vesicle will first dock, then prime, and wait to undergo exocytosis without any reverses in molecular stages. In fact, a central question is whether all vesicles that are docked are part of the RRP, or whether all RRP vesicles are docked (Kaeser and Regehr, 2017). We would suggest that, most likely, the process of vesicle docking to the presynaptic membrane and vesicles molecularly joining or leaving the RRP (e.g., priming and unpriming) is dynamic and that spontaneous and evoked fusion events occur depending on the state of vesicles in this one pool (Kobbersmed et al., 2020). This view is supported by a recent study that uses highly detailed electron microscopy measurements to demonstrate how docking and undocking can occur within 100 ms following stimulation (Kusick et al., 2020). In this way, the nanolocation and molecular controls of the two forms of synaptic transmission may be different while still allowing both types to be related to the number of vesicles participating in and populating the RRP. It is likely that there are both molecular and geographical changes that occur between docking and priming. There may also be instances of “superpriming” (Lee et al., 2013; Rothman et al., 2023), during which vesicles would engage in either spontaneous or evoked release as they move in and out of these states as part of a group of vesicles dynamically filling the RRP. It is worth noting that Munc13, a fundamental protein related to the first stages of vesicle docking, is necessary for supporting both spontaneous and evoked fusion (Varoqueaux et al., 2002; Quade et al., 2019; Tan et al., 2022), whereas RIM proteins that centrally control Pv but not docking only alter evoked release (Robinson et al., 2019; Brockmann et al., 2020; Zarebidaki et al., 2020). Moreover, this combined pool with multiple calcium sensors and states supports work from the calyx of Held, which has functionally supported a link between evoked and spontaneous fusion rates suggesting different calcium sensitivities and sensors from a single pool (Schneggenburger and Rosenmund, 2015). Taken together, this could indicate a fundamental connection between spontaneous release and formation of the RRP where evoked release is independent and can be molecularly refined at later stages of docking and priming.

### Pv is a presynaptic property that can alter evoked release independently of spontaneous release

The few studies that have explored the relationship between spontaneous and evoked release at single synapses have primarily focused on larger synapses with multiple AZs, such as the NMJ. A recent study in the vertebrate zebrafish NMJ using dual-labeling strategies of vesicle exocytosis and endocytosis found that spontaneous release was fueled by vesicles from the RRP (Egashira et al., 2022). However, studies at the *Drosophila* NMJ suggest that individual AZs specialize in either evoked or spontaneous release (Melom et al., 2013). More recently though, Grasskamp et al. (2023) also use calcium indicators in the *Drosophila* NMJ; however, they show that spontaneous release frequency acts as a predictor for the responsiveness of evoked release magnitude and suggests that the two have overlapping machinery when measured at fully mature release sites. In our study, we initially expected to see antagonistic relationships between evoked release magnitude and spontaneous release rates at the single synapse level, but instead found correlation. Our results would suggest that the developing NMJ is perhaps a rare case in specialized synapses. However, since Pv and RRP are independently regulated, there may be situations where spontaneous release rates are high, but evoked release is low, particularly during maturation of developing synapses in the NMJ. Interestingly, within the CNS, some synapses do contain large RRPs with very low Pv such as Purkinje cells which output to deep cerebellar nuclei neurons. Consistent with our results, recordings of miniature postsynaptic currents in deep cerebellar nuclei neurons show that Purkinje neurons have very high rates of spontaneous release compared to hippocampal boutons with small RRPs and higher Pvs (Mouginot and Gähwiler, 1995; Khan and Regehr, 2020). This fits well with our findings at the single synapse level of hippocampal neurons, where RRP is not only smaller but can also vary considerably from bouton to bouton. We would suggest that the NMJ may have very large variations in Pv at individual AZs, especially during development or in various cases of specialized muscle innervation. As such, spontaneous release may be regulated differently at the developing NMJ, perhaps by utilizing complexin as a control mechanism of docking and priming (Huntwork and Littleton, 2007; Jorquera et al., 2012; Wragg et al., 2013). Therefore, early findings of specialization at the NMJ do not contradict our findings, but in fact expose differences in Pv at AZs that could be masking RRP size differences. A great strength of the methods we developed here with iGluSnFR3 is that it provides a way to easily measure RRP size independent of evoked transmission and calcium. Excitingly, this technique can allow for quantification of the RRP at the level of individual synapses without resorting to a combination of modeling and inherent averaging from whole-cell electrophysiology recordings.

### Larger RRPs may be well positioned to potentially preserve synaptic connections through spontaneous release

We are most intrigued by the potential physiological purposes of regulating spontaneous release rates at individual synapses with dynamic changes in the size of the RRP. Ultrastructural EM studies suggest that the number of docked vesicles corresponds to spine size in hippocampal neurons (Bartol et al., 2015), and LTP-like protocols using super-resolution imaging also reveal changes in postsynaptic spines corresponding to changes in the number of vesicle docking sites (Tang et al., 2016). Our results would suggest that spontaneous release would be enhanced in these larger spines. Mechanistically, spontaneous release confers important information to the postsynaptic cell and local spine. One identified function of spontaneous vesicle release is to alter postsynaptic translation rates and to stabilize synapses (Sutton et al., 2004). Our findings that larger RRPs would enhance spontaneous release rates might explain why most imaging studies find that larger spines and postsynaptic densities are highly stable compared to smaller spines (Hill and Zito, 2013; Cane et al., 2014). Overall, it will be exciting to use iGluSnFR3 to help study these dynamics of evoked and spontaneous release in other preparations and to elucidate its role in spine morphology as well as mechanisms regulating spontaneous release and RRP size.

## Experimental Procedures

### Cell culture and transfection

Primary neurons from Sprague Dawley rats of either sex on postnatal day 0 were cultured for all experiments. Hippocampal CA1 to CA3 regions with the dentate gyri removed were harvested, tissue was dissociated into single cells with bovine pancreas trypsin, and cells were plated onto poly-l-ornithine-coated glass coverslips inside a 6-mm cloning cylinder as previously described (Hoppa et al., 2012). Ca^2+^ phosphate-mediated DNA transfection was performed on cultures at DIV 6. Cells were sparsely transfected with 3 µg of DNA using the following plasmids: iGluSnFR3 SGZ (#178330 on Addgene), mRuby synapsin (provided by de Juan-Sanz Laboratory (ICM-Paris Brain Institute)), and jGCaMP8f (#162376 on Addgene). DNA was precipitated in minimal essential media at a 1:10 ratio, applied to cells, and incubated for 8 min. All experiments were performed on mature neurons between 14 and 20 DIV. To ensure reproducibility, experiments were performed on neurons from a minimum of three separate cultures. All protocols used were approved by the Institutional Animal Care and Use Committee at Dartmouth College and conform to the NIH Guidelines for the Care and Use of Animals.

### Live cell imaging

All experiments were performed at 35°C using a custom-built objective heater. Cultured cells were mounted in a rapid-switching laminar flow perfusion and stimulation chamber on the stage of a custom-built epifluorescence laser microscope. The total volume of the imaging chamber was 100 μl and neurons were perfused at a rate of 400 μl/min in a modified Tyrode's solution containing the following (in mM): 119 NaCl, 2.5 KCl, 1.3 CaCl_2_, 2.7 MgCl_2_, 25 HEPES, and 30 glucose with 10 μM cyanquixaline (Sigma-Aldrich) and 50 μM APV (Sigma-Aldrich). A subset of recordings were made with the addition of 100 µM TTX (Tocris). Images were obtained using a Zeiss Observer Z1 equipped with an EC Plan-Neofluar 40× 1.3 numerical aperture (NA) oil immersion objective. All images were captured with an ORCA-Fusion Digital CMOS camera (Hamamatsu). All excitation light occurred using solid-state illumination via SPECTRA light engine (Lumencor).

### Immunohistochemistry

For immunohistochemistry, primary cultures were fixed in 4% PFA/4% sucrose in PBS for 15 min and washed in PBS (3×). Cultures were then permeabilized with 0.2% Triton X-100, washed in PBS (3×), and blocked in 5% goat serum/5% bovine serum albumin (1:1) for 30 min. Cultures were incubated overnight at 4°C in anti-GFP, chicken IgY at a 1:1,000 dilution (Invitrogen; A10262). The following day, cultures were washed in PBS (3×) and incubated for 1 h at room temperature in Alexa Fluor 488 goat antichicken IgG at a 1:1,000 dilution (Life Technologies; A11039) diluted in 5% goat serum in PBS. After a final set of PBS washes (3×), coverslips were mounted for imaging.

### Fixed cell imaging

Fixed cells were imaged on a Nikon Ti2-inverted spinning disk confocal microscope using a 40× 1.3 NA oil immersion objective, excited using 460 nm wavelength and imaged with 100 ms exposure time. *Z*-stack with 0.3 mm steps were taken of an individual cell. Cell was also captured using transmitted light in the brightfield channel. *Z*-stack was projected using ImageJ (NIH).

### Evoked release measurements

iGluSnFR3 fluorescence was recorded with 40 ms exposure time; images were acquired at 25 Hz. Cells were illuminated by a 475/28 nm diode at 10.2 mW. APs were evoked by passing 1 ms current pulses yielding fields of ∼12 V/cm^2^ via platinum iridium electrodes. The average DF/F amplitude of 20 individual APs was used to calculate evoked release amplitude for each individual bouton.

### Spontaneous release measurements

iGluSnFR3 fluorescence was recorded as described above. Spontaneous release frequencies were quantified over the course of 2 min of recording time. Spontaneous release events were selected by using the MATLAB peaks function (MathWorks) and a threshold of three standard deviations above the baseline fluorescence.

### Bouton selection

All experiments were transfected with synapsin-mRuby to identify presynaptic boutons. Only cells expressing both synapsin-mRuby and iGluSnFR3 or jGCaMP8f were imaged. ROIs were selected for the synapsin channel by applying a maximum entropy threshold to images. Boutons that fell within the threshold and were in the plane of focus were evaluated further. Boutons that fell on axonal branch points or overlapped with another axon were discarded. In the case where boutons were within 0.8 mm of one another (i.e., their ROI boundaries were overlapping), one of the two boutons was arbitrarily selected and the other was discarded. After applying these criteria, 88% of the total number of boutons could be measured per cell. Boutons fulfilling these requirements were measured using a 1.625 × 1.625 mm ROI centered on the brightest pixel. All ROI measurements were taken using ImageJ (NIH).

### Synapsin quantification

Synapsin presence was identified using an mRuby synapsin tag. Synapsin intensity was quantified by taking the average fluorescent intensity of a 1.625 × 1.625 mm ROI centered on the brightest pixel as measured using ImageJ (NIH).

### Sucrose measurements

Sucrose measurements were acquired using the same imaging conditions as described above. After a baseline recording of 20 s had been acquired, 25 ml of Tyrode's solution containing 1.5 mM CaCl_2_ and 2.5 mM MgCl_2_ with the addition of 500 mM sucrose was dispensed into the imaging chamber via laminar flow at a rate of 2.5 ml/s using an Eppendorf Repeater stream pipette. The AUC of the iGluSnFR3 signal during the 20 s following sucrose administration was used to quantify the size of the RRP. Calculations were done in MATLAB (MathWorks) using the trapezoidal numerical integration function.

### Roscovitine measurements

Neurons were treated with roscovitine (Alomone Labs), administered in Tyrode's solution at 100 mM, for 10 min before evoked and spontaneous measurements were acquired.

### WIN measurements

Neurons were treated with WIN 55,212-2 mesylate salt (WIN) (Sigma-Aldrich), administered in Tyrode's solution at 1 mM, for 15 min before evoked and spontaneous measurements were acquired.

### PDBu measurements

Neurons were treated with PDBu (Tocris), administered in Tyrode's solution at 1 mM, for 5 min before evoked and spontaneous measurements were acquired.

### High-frequency stimulation measurements

Neurons were stimulated with 10 APs at 100 Hz over five trials. The average AUC of the change in fluorescence evoked by the 10 APs was calculated using the trapezoidal numerical integration functions in MATLAB (MathWorks).

### jGCaMP8f measurements

Spontaneous and evoked release in jGCaMP8f and synapsin-mRuby transfected neurons were evaluated using the same parameters as listed above. Spontaneous activity was recorded for 2 min. Evoked release was measured by stimulating neurons with 20 individual APs at a rate of 0.5 Hz. Spontaneous recordings were evaluated by taking the average amplitude of evoked events at an individual bouton and searching for events of equal or greater amplitude in the spontaneous activity recordings. No events equaling the same or greater amplitude of an evoked event were found at any bouton in all of the spontaneous activity recordings.

### Conotoxin (CnTX) measurements

Neurons were treated with ω-conotxin GVIA (Tocris) administered in Tyrode's solution at 1 mM for 5 min before evoked and spontaneous measurements were acquired.

### Agatoxin (AgTX) measurements

Neurons were treated with ω-agatoxin IVA (Tocris) administered in Tyrode's solution at 500 nM for 2 min before evoked and spontaneous measurements were acquired.
